# ﻿The Dolichens database: the lichen biota of the Dolomites

**DOI:** 10.3897/mycokeys.103.115462

**Published:** 2024-03-11

**Authors:** Luana Francesconi, Matteo Conti, Gabriele Gheza, Stefano Martellos, Pier Luigi Nimis, Chiara Vallese, Juri Nascimbene

**Affiliations:** 1 BIOME Lab, Alma Mater Studiorum - University of Bologna, Bologna, Italy Alma Mater Studiorum - University of Bologna Bologna Italy; 2 Department Of Life Sciences, University of Trieste, Trieste, Italy University of Trieste Trieste Italy; 3 Department Of Earth, Environmental and Life Sciences, University of Genova, Genova, Italy University of Genova Genova Italy

**Keywords:** Georeferencing, herbarium specimens, historical records, lichen diversity, occurrence, open inventory

## Abstract

The Dolichens project provides the first dynamic inventory of the lichens of the Dolomites (Eastern Alps, Italy). Occurrence records were retrieved from published and grey literature, reviewed herbaria, unpublished records collected by the authors, and new sampling campaigns, covering a period from 1820 to 2022. Currently, the dataset contains 56,251 records, referring to 1,719 infrageneric taxa, reported from 1820 to 2022, from hilly to nival belts, and corresponding to about half of the species known for the whole Alpine chain. Amongst them, 98% are georeferenced, although most of them were georeferenced a posteriori. The dataset is available through the Global Biodiversity Information Facility (GBIF; https://www.gbif.org/es/dataset/cea3ee2c-1ff1-4f8e-bb37-a99600cb4134) and through the Dolichens website (https://italic.units.it/dolichens/). We expect that this open floristic inventory will contribute to tracking the lichen diversity of the Dolomites over the past 200 years, and providing the basis for future taxonomic, biogeographical, and ecological studies.

## ﻿Introduction

The Dolomites, in the Southeastern Italian Alps, were declared a UNESCO World Heritage Site in June 2009, because of the uniqueness of their geology and landscapes. Such a variety of spectacular forms are related to their complex geological origin, as well as to the processes that have modeled the landscape (Messner and Tappeiner 2010). The Dolomites, along with the surrounding areas, constitute a complex mosaic of habitats, which underpin their high biodiversity (Pignatti and Pignatti 2016; Rota et al. 2022).

The Dolomites are one of the lichenologically best-known areas in Italy. Here, lichen diversity is strictly related to the variety of climatic conditions and substrates (Nimis 1993). Trentino-Alto Adige, in particular, is the lichenologically richest administrative region of Italy, with 1,684 infrageneric taxa reported to date, while 1,234 and 1,364 infrageneric taxa are known for the neighboring Veneto and Lombardia regions (Nimis and Martellos 2024).

These comparatively high figures are due to the long tradition of lichenological research in the area, which has been explored since the 19^th^ century. The majority of historical contributions are attributed to Ferdinand Arnold (1828–1901), who surveyed South Tyrol in the last years of the 1800s and published the results in his Lichenologische Ausfluge in Tirol (1868–1897). Another relevant contribution was provided by Ernst Kernstock (1852–1900) in his Lichenologische Beitrage (1890–1896). The lichen records collected in the area until 1901 were summarized by Dalla Torre and Sarnthein (1902) in one of the oldest “checklists”. In the first half of the 20^th^ century, these territories were mainly explored by Pio Bolzon (1867–1940) and Maria Cengia Sambo (1888–1939), who published several contributions on local lichen flora (Nimis 1993).

Despite the lichenological relevance of the Dolomites, no modern synthesis of their lichen diversity was ever attempted. There are important resources on the lichens of the Alps (Nimis et al. 2018), and on the lichen biotas of Italian Alpine regions (Martellos et al. 2023), but neither of these resources recognizes the Dolomites as an independent operational geographic unit. In addition, to our knowledge, no geo-referenced occurrence records of lichens are available online for the Dolomites.

Inventories based on new field explorations (Vondrák et al. 2016; Spribille et al. 2020), review of herbarium specimens, and literature records (Isocrono et al. 2007; Himelbrant et al. 2018) provide fundamental information on ecology and distribution of species. Furthermore, they foster taxonomic discoveries, including the description of new species and higher groups (Spribille et al. 2020; Leavitt et al. 2021; Nascimbene et al. 2022). Finally, if occurrence records are geo-referenced, it is possible to perform spatial analyses, such as biogeographical studies or distribution modeling (Bloom et al. 2018; Guttová et al. 2019; Marsico et al. 2020), which are pivotal for revealing biodiversity patterns, predicting potential shifts in a global change scenario and contributing to effective conservation guidelines (Ellis et al. 2007; Eaton et al. 2018; Nelsen and Lumbsch 2020).

The Dolichens project was launched in 2022. It aims at building a dynamic geo-referenced inventory of the lichen biota of the Dolomites by aggregating occurrence records from published and gray literature (such as university research theses), unpublished data, and herbaria from the 19^th^ century onwards. The project aims at aggregating occurrence records from recent surveys as well. The result is a database accessible online (https://italic.units.it/dolichens/), which is continuously updated.

## ﻿Sampling methods

**Figure 1.**
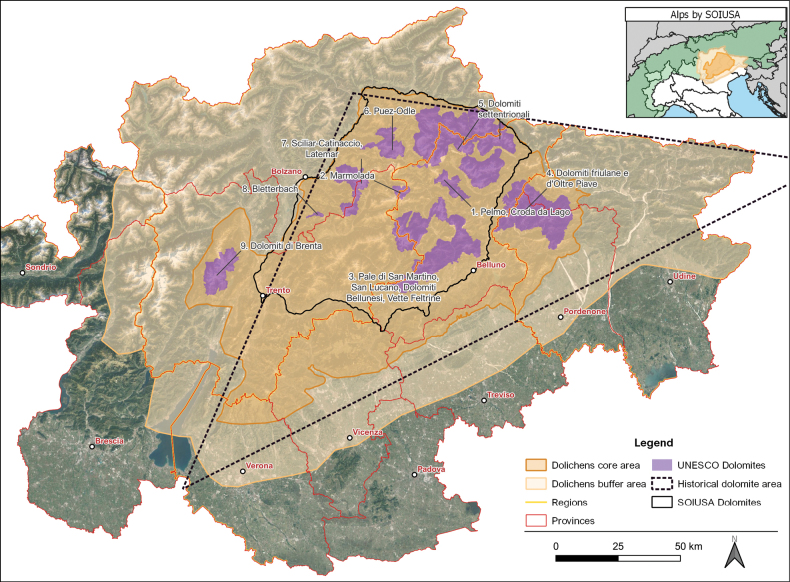
Study area of the project. The core area (dark orange) is wider than the SOIUSA definition (black line, Marazzi 2005). It includes all the 9 UNESCO systems (purple) and it extends southward to the Prealps including the Little Dolomites as well, retracing the historical definition (dotted black line, Bosellini 1989).

**Description**: The Dolichens system hosts georeferenced occurrence records of lichens in the Dolomites, from the 19^th^ century onwards. The geographical delimitation of the Dolomites region was, however, not simple, since several contrasting definitions exist (Bentivoglio and De Simoni 1980; Bosellini 1989; Marazzi 2005; Messner and Tappeiner 2010). In this study, a core area was selected (Fig. 1), which is wider than the most common geographical definition for the Dolomites provided by the Partition of the Alps and the SOIUSA (Marazzi 2005). It spans an area of 11,735 km^2^, encompassing the territories surrounding the 9 UNESCO systems (Messner and Tappeiner 2010), thereby incorporating the Friulian and Brenta Dolomites, extending southward to the Venetian and Carnic Prealps, in order for us to be able to include the area of the “Little Dolomites” (Bosellini 1989) as well. In addition, a buffer area was delimited (Fig. 1), which extends northward and eastward to the national borders, enclosing the Stelvio National Park and the Adamello Brenta Natural Park on the west, and reaching the border of the Po-Valley near Verona, covering a surface of 29,299 km^2^. Data aggregation was focused on the core area, while data from the buffer area were aggregated when they were included in the same resource reporting data from the core area. Thus, the Dolichens project provides a preliminary baseline for the development of a future lichen inventory of the Eastern Italian Alps.

**Sampling description**: For this study occurrence records were collected from different sources. First of all, to compile a baseline inventory of the lichens of the Dolomites, we gathered records from literature starting from the 19^th^ century (such as checklists, vegetation surveys, and taxonomic revisions). Among them, the most exhaustive historical source is the catalogue of Dalla Torre and Sarnthein (1902), from which 10,299 records referring to 885 taxa were retrieved. Furthermore, unpublished records collected by the authors, as well as from gray literature, were gathered. Herbarium specimen data were retrieved from historical herbaria, some of which were reviewed by the authors of this contribution, such as those of Alberto Parolini (1788–1867), Francesco Ambrosi (1821–1897), or Giacomo Bresadola (1847–1929). Finally, sampling campaigns were carried out and are still ongoing, mainly in the Paneveggio Pale di San Martino Natural Park, the Adamello Brenta Natural Park, and the Dolomiti Bellunesi National Park. They are focused on protected areas and allow the investigation of both historical sites and new locations, which have been poorly explored by lichenologists.

**Quality control**: Specimens were collected and identified/revised by the experienced lichenologists of our group (e.g. Nascimbene, Nimis) and colleagues from other universities. However, the current database is not a critical checklist. The latter will be developed in the future by critically evaluating all the collected records.

Scientific names were automatically aligned to the latest version of the annotated checklist of Italian lichens (Nimis 2016), available on ITALIC 7.0 (Martellos et al. 2023) by means of a customized version of the FlorItaly name-matching tool (Conti et al. 2021). Then, the scientific names were normalized against the GBIF backbone (GBIF Secretariat 2023). The verbatim names have been always retained together with the currently accepted name.

When geographical coordinates of the collection locality were missing, the records were georeferenced a posteriori using Google Maps, Google Earth, and regional GIS maps, following the best practices proposed by Chapman and Wieczorek (2020).

## ﻿Geographic coverage

**Figure 2.**
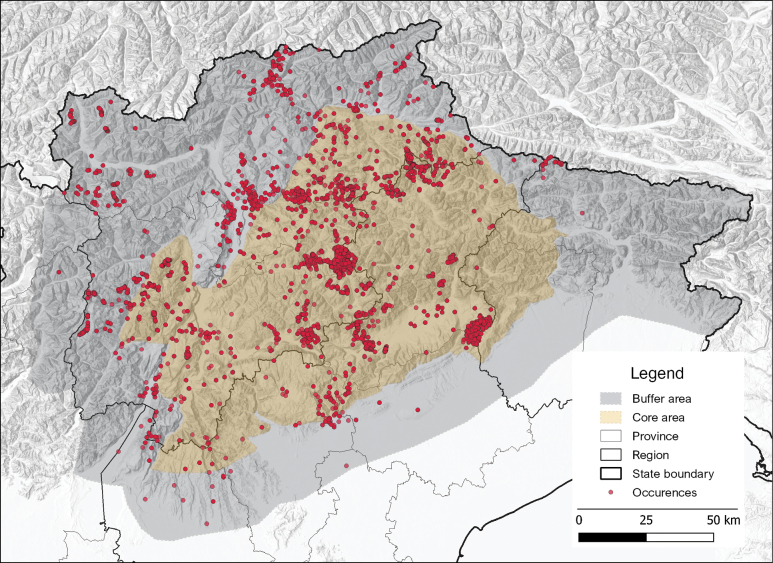
Distribution map of the Dolichens database occurrences and specimens in the study area. Map created using the Free and Open Source QGIS

**Description**: The dataset contains occurrence data of lichens reported for 4 administrative regions and 11 provinces of Italy: Friuli Venezia Giulia (Udine 366 and Pordenone 410), Lombardy (Brescia 11 and Sondrio 2), Trentino Alto Adige (Trento 18,052 and Bolzano 20,543), Veneto (Belluno 14,270, Padova 1, Treviso 779, Verona 106 and Vicenza 325). The distribution of records in the study area is shown in Fig. 2.

**Coordinates**: 10.2782 and 13.7173 Latitude; 45.3834 and 47.0918 Longitude.

## ﻿Taxonomic coverage

**Description**: According to the GBIF Taxonomic Backbone, the dataset comprises taxa from 39 orders, 102 families, and 416 genera.

The following families are represented: Acarosporaceae, Adelococcaceae, Arctomiaceae, Arthoniaceae, Arthopyreniaceae, Arthrorhaphidaceae, Ascodichaenaceae, Baeomycetaceae, Biatorellaceae, Byssolomataceae, Caliciaceae, Candelariaceae, Catillariaceae, Chrysotrichaceae, Cladoniaceae, Coccocarpiaceae, CoenogoniaceaeCollemataceae, Coniocybaceae, Cystocoleaceae, Dacampiaceae, Didymellaceae, Elixiaceae, Epigloeaceae, Fuscideaceae, Gomphillaceae, Graphidaceae, Gyalectaceae, Haematommataceae, Helocarpaceae, Hygrophoraceae, Hymeneliaceae, Hysteriaceae, Icmadophilaceae, Lecanographaceae, Lecanoraceae, Lecideaceae, Leprocaulaceae, Lichenotheliaceae, Lichinaceae, Lobariaceae, Lopadiaceae, Malmideaceae, Massalongiaceae, Megasporaceae, Melaspileaceae, Microcaliciaceae, Monoblastiaceae, Mycocaliciaceae, Mycoporaceae, Mycosphaerellaceae, Myriangiaceae, Naetrocymbaceae, Nephromataceae, Ochrolechiaceae, Opegraphaceae, Ophioparmaceae, Pannariaceae, Parmeliaceae, Patellariaceae, Peltigeraceae, Peltulaceae, Pertusariaceae, Phlyctidaceae, Physciaceae, Placynthiaceae, Polycoccaceae, Porinaceae, Porpidiaceae, Psilolechiaceae, Psoraceae, Pycnoraceae, Pyrenulaceae, Ramalinaceae, Ramboldiaceae, Rhizocarpaceae, Roccellaceae, Sagiolechiaceae, Sareaceae, Sarrameanaceae, Schaereriaceae, Sclerococcaceae, Scoliciosporaceae, Sphaerophoraceae, Sphinctrinaceae, Sporastatiaceae, Stereocaulaceae, Stictidaceae, Strangosporaceae, Strigulaceae, Teloschistaceae, Tephromelataceae, Thelenellaceae, Thelocarpaceae, Trapeliaceae, Trypetheliaceae, Umbilicariaceae, Vahliellaceae, Varicellariaceae, Verrucariaceae, Xanthopyreniaceae, and Xylographaceae.

**Figure 3.**
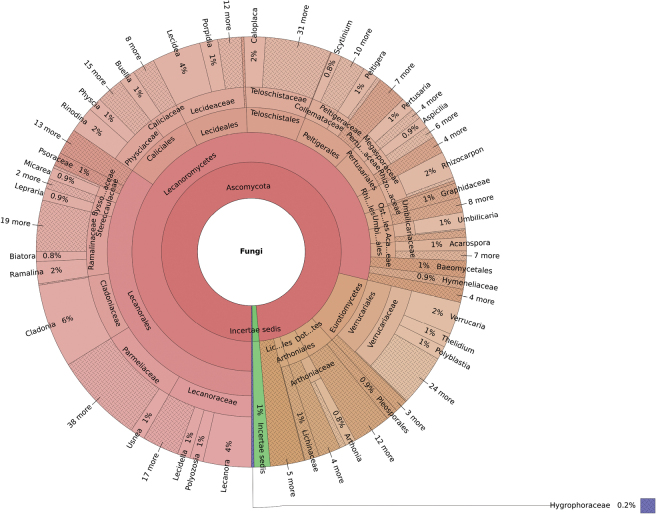
Relative abundance at the genus level referred to the total number of taxa (1994), created using the Krona graph tool (Ondov et al. 2011).

**Figure 4.**
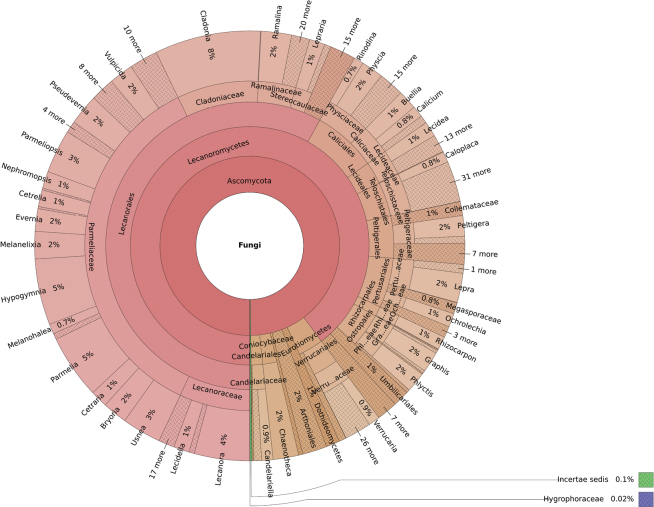
Relative abundance at the genus level referred to the total number of occurrences (56251), created using the Krona graph tool (Ondov et al. 2011).

Taxonomic distribution (Fig. 3) and distribution of occurrences (Fig. 4) among each kingdom, phylum, class, order, family, and genus can be graphically visualized as a Krona graph, an interactive multi-layered pie chart that allows the exploration of hierarchical data (the interactive file is provided in Suppl. material 1).

## ﻿Temporal coverage

**Figure 5.**
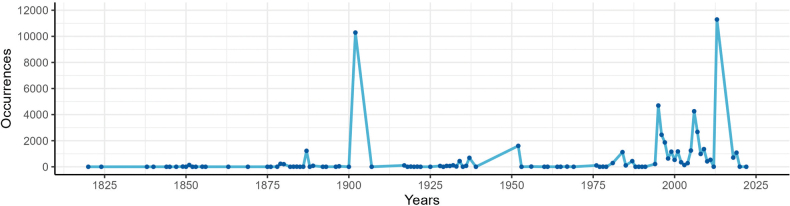
Lichen occurrences of the Dolichens dataset per year.

Data were reported from 1820 to 2022. Occurrences per year are shown in Fig. 5. The highest number of occurrences in 1902 corresponds to the data retrieved from the most exhaustive historical source, the catalogue of Dalla Torre and Sarnthein (1902), which collects data derived from the publications of previous authors, like Ferdinand Arnold, who explored the Tyrol during the 19^th^ century. Then, the more recent peaks of occurrences from the 1990s correspond to the newfound national interest in lichenology, and the lichenological exploration by the research group of the University of Bologna.

## ﻿Usage licence

This work is licensed under a Creative Commons Attribution (CC-BY) 4.0 License.

## ﻿Data resources

**Data package title**: Dolichens project: the lichen biota of the Dolomites.

**Resource link**: https://doi.org/10.15468/64sy7b.

**Alternative identifiers**: https://cloud.gbif.org/eca/resource?r=dolichens_project.

**Number of data sets**: 1.

**Data set name**: Dolichens project: the lichen biota of the Dolomites.

**Download URL**: https://www.gbif.org/occurrence/download?dataset_key=cea3ee2c-1ff1-4f8e-bb37-a99600cb4134.

**Data format**: Darwin Core.

**Description**: Launched in 2022 the Dolichens project aims to assemble an inventory of the lichens reported from the Dolomite area since the 19^th^ century. Data of lichen occurrences have been retrieved and aggregated from herbaria, literature, unpublished data, and new sampling campaigns.

**Table:** 

Column label	Column description
occurrenceID	A unique identifier for the occurrence.
basisOfRecord	The source or nature of the record.
verbatimIdentification	The original, unaltered identification of the organism.
scientificName	The scientifically accepted name of the organism.
Kingdom	The taxonomic kingdom to which the organism belongs.
eventDate	The date when the occurrence was observed or recorded.
stateProvince	The administrative region in which the organism was recorded.
County	The name of the next smaller administrative region than state Province in which the organism was recorded.
Locality	The location where the organism was observed.
decimalLatitude	The latitude coordinates of the occurrence in decimal format.
decimalLongitude	The longitude coordinates of the occurrence in decimal format.
geodeticDatum	The reference geodetic datum for the coordinate data.
coordinateUncertaintyInMeters	The level of uncertainty associated with the geographic coordinates, in meter
minimumElevationInMeters	The lowest elevation at which the organism was found.
maximumElevationInMeters	The highest elevation at which the organism was found.
Continent	The continent where the occurrence was recorded.
Country	The country where the occurrence was recorded.
countryCode	The code of the country where the occurrence was recorded.
recordedBy	The person responsible for recording the occurrence.
identifiedBy	The person responsible for identifying the organism.
associatedReferences	References or sources of information associated with the occurrence.
License	The terms and conditions under which data can be used and shared
Language	The language in which the data or metadata for the occurrence is written

## ﻿Additional information

### Author ORCIDs

Luana Francesconi https://orcid.org/0000-0003-1745-7069

Matteo Conti https://orcid.org/0009-0003-4917-2639

Stefano Martellos https://orcid.org/0000-0001-5201-8948

Pier Luigi Nimis https://orcid.org/0000-0003-3523-0183

Chiara Vallese https://orcid.org/0000-0002-8531-5954

Juri Nascimbene https://orcid.org/0000-0002-9174-654X

### Data availability

All of the data that support the findings of this study are available in the main text or Supplementary Information.

## Supplementary materials

Supplementary material 1 Krona graph taxa and occurrences Data type html Explanation note Interactive file of the krona graph, showing relative abundance of the total taxa and the total occurrences in in each class, order, family and genus. https://binary.pensoft.net/file/1000092This dataset is made available under the Open Database License (http://opendatacommons.org/licenses/odbl/1.0/). The Open Database License (ODbL) is a license agreement intended to allow users to freely share, modify, and use this Dataset while maintaining this same freedom for others, provided that the original source and author(s) are credited.Luana Francesconi, Matteo Conti

## References

[B1] Arnold F (1869) Lichenologische Ausflüge in Tirol. IV. Der Schlern. Zoologisch-Botanische Gesellschaft, Wien, 606–656.

[B2] Arnold F (1871) Lichenologische Ausflüge in Tirol. VI. Der Waldrast. Zoologisch-Botanische Gesellschaft, Wien, 1103–1148.

[B3] Arnold F (1874) Lichenologische Ausflüge in Tirol. XIII. Der Brenner. Zoologisch-Botanische Gesellschaft, Wien, 231–284.

[B4] Arnold F (1876) Lichenologische Ausflüge in Tirol. XVI. Ampezzo. Zoologisch-Botanische Gesellschaft, Wien, 389–414.

[B5] Arnold F (1879) Lichenologische Ausflüge in Tirol. XX. Predazzo. Zoologisch-Botanische Gesellschaft, Wien, 351–394.

[B6] Arnold F (1886) Lichenologische Ausflüge in Tirol. XXII. Sulden. Zoologisch-Botanische Gesellschaft, Wien, 61–88.

[B7] Arnold F (1887) Lichenologische Ausflüge in Tirol. XXII. Predazzo und Paneveggio. Zoologisch-Botanische Gesellschaft, Wien, 81–150.

[B8] Arnold F (1896) Lichenologische Ausflüge in Tirol. XXVI. Pians. XXVII. Galtür. XXVIII. Wolkenstein. XXIX. Plansee. Zoologisch-Botanische Gesellschaft, Wien, 1–43.

[B9] Bentivoglio G De Simoni G (1980) Partizione delle Alpi (in 220 gruppi). Tipografia Alzani, Pinerolo.

[B10] Bloom TDS Flower A DeChaine EG (2018) Why georeferencing matters: Introducing a practical protocol to prepare species occurrence records for spatial analysis. Ecology and Evolution 8: 765–777. 10.1002/ece3.3516 29321912 PMC5756859

[B11] Bosellini A (1989) La storia geologica delle Dolomiti. Ed. Dolomiti, 148 pp.

[B12] Chapman AD Wieczorek JR (2020) Georeferencing best practices. Version 1.0. GBIF Secretariat. [Report] 10.15468/doc-gg7h-s853

[B13] Conti M Nimis PL Martellos S (2021) Match Algorithms for Scientific Names in FlorItaly, the Portal to the Flora of Italy. Plants 10: 974. 10.3390/plants10050974 PMC8153551 34068389

[B14] Dalla Torre KW Sarnthein LG (1902) Die Flechten (Lichenes) von Tirol, Vorarlberg und Liechtenstein. In: K.W. Dalla Torre, Sarnthein LG (Eds) Flora der gefürsteten Grafschaft Tirol, des Landes Vorarlberg und des Fürstenthumes Liechtenstein. Nach eigenen und fremden Beobachtungen, Sammlungen und den Litteraturquellen. Verlag der Wagner’-schen Universitäts-Buchhandlung, Innsbruck, 1–693.

[B15] Eaton S Ellis C Genney D Thompson R Yahr R Haydon DT (2018) Adding small species to the big picture: Species distribution modelling in an age of landscape scale conservation. Biological Conservation 217: 251–258. 10.1016/j.biocon.2017.11.012

[B16] Ellis CJ Coppins BJ Dawson TP Seaward MRD (2007) Response of British lichens to climate change scenarios: Trends and uncertainties in the projected impact for contrasting biogeographic groups. Biological Conservation 140: 217–235. 10.1016/j.biocon.2007.08.016

[B17] GBIF Secretariat (2023) GBIF Backbone Taxonomy. 10.15468/39OMEI

[B18] Guttová A Fačkovcová Z Martellos S Paoli L Munzi S Pittao E Ongaro S (2019) Ecological specialization of lichen congeners with a strong link to Mediterranean-type climate: a case study of the genus *Solenopsora* in the Apennine Peninsula. The Lichenologist 51: 75–88. 10.1017/S0024282918000543

[B19] Himelbrant DE Stepanchikova IS Kuznetsova ES Motiejūnaitė J Konoreva LA (2018) Konevets Island (Leningrad Region, Russia) – a historical refuge of lichen diversity in Lake Ladoga. Folia Cryptogamica Estonica 55: 51–78. 10.12697/fce.2018.55.07

[B20] Isocrono D Matteucci E Ferrarese A Pensi E Piervittori R (2007) Lichen colonization in the city of Turin (N Italy) based on current and historical data. Environmental Pollution 145: 258–265. 10.1016/j.envpol.2006.03.031 16777296

[B21] Kernstock E (1890a) Lichenologische Beiträge I. Pinzolo (Südtirol). Zoologisch-Botanische Gesellschaft, Wien 40, 317–339.

[B22] Kernstock E (1890b) Lichenologische Beiträge II. Bozen. Zoologisch-Botanische Gesellschaft, Wien 40, 339–350.

[B23] Kernstock E (1891) Lichenologische Beiträge III. Jenesien bei Bozen. Zoologisch-Botanische Gesellschaft, Wien 41, 701–738.

[B24] Kernstock E (1892a) Lichenologische Beiträge. IV. M. Gazza (Paganella, 2120 m) in Südtirol. Zoologisch-Botanische Gesellschaft, Wien 42, 319–325.

[B25] Kernstock E (1892b) Lichenologische Beiträge. V. Judicarien. Zoologisch-Botanische Gesellschaft, Wien 42, 325–349.

[B26] Kernstock E (1894) Lichenologische Beiträge. VI. Möltener Alpen. Zoologisch-Botanische Gesellschaft, Wien 44, 191–224. 10.1007/BF01790222

[B27] Kernstock E (1896) Lichenologische Beiträge. VII. Ehrenburg im Pusterthale. Zoologisch-Botanische Gesellschaft, Wien 46, 279–310.

[B28] Leavitt SD Hollinger J Summerhays S Munger I Allen J Smith B (2021) Alpine lichen diversity in an isolated sky island in the Colorado Plateau, USA—Insight from an integrative biodiversity inventory. Ecology and Evolution 11: 11090–11101. 10.1002/ece3.7896 34429905 PMC8366874

[B29] Marazzi S (2005) Atlante orografico delle Alpi: SOIUSA : suddivisione orografica internazionale unificata del sistema alpino. Priuli & Verlucca, 422 pp.

[B30] Marsico TD Krimmel ER Carter JR Gillespie EL Lowe PD McCauley R Morris AB Nelson G Smith M Soteropoulos DL Monfils AK (2020) Small herbaria contribute unique biogeographic records to county, locality, and temporal scales. American Journal of Botany 107: 1577–1587. 10.1002/ajb2.1563 33217783 PMC7756855

[B31] Martellos S Conti M Nimis PL (2023) Aggregation of Italian Lichen Data in ITALIC 7.0. Journal of Fungi 9: 556. 10.3390/jof9050556 PMC10219532 37233266

[B32] Messner R Tappeiner G (2010) Dolomiti. Patrimonio dell’umanità. Mondadori Electa, 268 pp.

[B33] Nascimbene J Gheza G Bilovitz PO Francesconi L Hafellner J Mayrhofer H Salvadori M Vallese C Nimis PL (2022) A hotspot of lichen diversity and lichenological research in the Alps: the Paneveggio-Pale di San Martino Natural Park (Italy). MycoKeys 94: 37–50. 10.3897/mycokeys.94.95858 36760543 PMC9836431

[B34] Nelsen MP Lumbsch HT (2020) A data-driven evaluation of lichen climate change indicators in Central Europe. Biodiversity and Conservation 29: 3959–3971. 10.1007/s10531-020-02057-8

[B35] Nimis P (2016) The Lichens of Italy. A Second Annotated Catalogue. EUT Edizioni Università di Trieste.

[B36] Nimis PL (1993) The lichens of Italy: an annotated catalogue. Museo regionale di scienze naturali, Torino, 897 pp.

[B37] Nimis PL Martellos S (2024) ITALIC - The Information System on Italian Lichens. Version 7.0. [Available from:] https://italic.units.it/

[B38] Nimis PL Hafellner J Roux C Clerc P Mayrhofer H Martellos S Bilovitz PO (2018) The lichens of the Alps – an annotated checklist. MycoKeys 31: 1–634. 10.3897/mycokeys.31.23568 PMC5914158 29706791

[B39] Ondov BD Bergman NH Phillippy AM (2011) Interactive metagenomic visualization in a Web browser. BMC Bioinformatics 12: 385. 10.1186/1471-2105-12-385 PMC3190407 21961884

[B40] Pignatti E Pignatti S (2016) Plant Life of the Dolomites: Atlas of Flora. Springer, 495 pp. 10.1007/978-3-662-48032-8

[B41] Rota F Casazza G Genova G Midolo G Prosser F Bertolli A Wilhalm T Nascimbene J Wellstein C (2022) Topography of the Dolomites modulates range dynamics of narrow endemic plants under climate change. Scientific Reports 12: 1398. 10.1038/s41598-022-05440-3 PMC8792058 35082360

[B42] Spribille T Fryday AM Pérez-Ortega S Svensson M Tønsberg T Ekman S Holien H Resl P Schneider K Stabentheiner E Thüs H Vondrák J Sharman L (2020) Lichens and associated fungi from Glacier Bay National Park, Alaska. The Lichenologist 52: 61–181. 10.1017/S0024282920000079 32788812 PMC7398404

[B43] Vondrák J Malíček J Palice Z Coppins B Kukwa M Czarnota P Sanderson N Acton A (2016) Methods for obtaining more complete species lists in surveys of lichen biodiversity. Nordic Journal of Botany 34: 619–626. 10.1111/njb.01053

