# Supplementary material for: ﻿The Dolichens database: the lichen biota of the Dolomites
Source: MycoKeys. 2024 Mar 11;103:25–35. doi: 10.3897/mycokeys.103.115462 (PMC10948996; doi:10.3897/mycokeys.103.115462)
Supplement: Supplementary material 1 — Krona graph taxa and occurrences [file mycokeys-103-025-s001.html]

Javascript must be enabled to view this page.

magnitude

Dolichens taxa
Dolichens occurrences

 1994
 56251

 1994
 56251

 1966
 56161

 58
 1031

 58
 1031

 25
 246

 16
 219

 2
 8

 2
 6

 1
 8

 2
 3

 1
 1

 1
 1

 2
 428

 2
 428

 6
 33

 3
 21

 2
 3

 1
 9

 11
 89

 1
 2

 9
 86

 1
 1

 14
 235

 1
 9

 1
 2

 1
 1

 2
 19

 1
 2

 2
 178

 1
 1

 3
 20

 1
 2

 1
 1

 14
 877

 14
 877

 11
 807

 2
 323

 9
 484

 3
 70

 3
 70

 15
 1076

 15
 1076

 15
 1076

 15
 1076

 75
 577

 1
 6

 1
 6

 1
 6

 1
 8

 1
 8

 1
 8

 14
 103

 14
 103

 14
 103

 1
 6

 1
 6

 1
 6

 8
 19

 8
 19

 2
 7

 1
 2

 5
 10

 3
 13

 3
 13

 3
 13

 8
 102

 8
 102

 4
 11

 2
 78

 2
 13

 1
 5

 1
 5

 1
 5

 1
 1

 1
 1

 1
 1

 2
 10

 2
 10

 1
 1

 1
 9

 18
 220

 1
 1

 1
 1

 2
 80

 1
 47

 1
 33

 2
 3

 2
 3

 2
 2

 2
 2

 11
 134

 4
 24

 6
 94

 1
 16

 2
 8

 2
 8

 2
 8

 15
 76

 2
 4

 2
 4

 13
 72

 9
 51

 1
 1

 3
 20

 188
 1918

 12
 147

 9
 144

 5
 113

 1
 15

 1
 5

 2
 11

 3
 3

 3
 3

 6
 159

 6
 159

 1
 4

 5
 155

 1
 1

 1
 1

 1
 1

 169
 1611

 1
 2

 1
 2

 1
 4

 1
 4

 167
 1605

 4
 49

 1
 5

 9
 94

 8
 103

 8
 169

 3
 29

 1
 1

 2
 6

 3
 9

 1
 1

 1
 2

 1
 3

 1
 1

 3
 8

 2
 173

 3
 35

 2
 21

 1
 3

 20
 158

 1
 1

 4
 4

 2
 7

 13
 108

 21
 113

 49
 482

 1
 1

 2
 19

 17
 41

 14
 38

 14
 38

 13
 37

 1
 1

 3
 3

 3
 3

 1
 1

 2
 2

 1571
 50487

 35
 289

 35
 289

 20
 183

 1
 3

 3
 21

 1
 1

 1
 5

 5
 23

 3
 38

 1
 15

 1
 2

 1
 2

 1
 2

 25
 303

 7
 66

 2
 10

 4
 50

 1
 6

 14
 193

 1
 19

 1
 1

 1
 1

 1
 4

 3
 55

 2
 10

 1
 14

 4
 89

 4
 44

 1
 4

 1
 1

 2
 39

 158
 4044

 58
 1630

 2
 55

 4
 173

 24
 817

 13
 472

 1
 23

 1
 7

 9
 58

 1
 6

 3
 19

 100
 2414

 5
 155

 1
 7

 1
 33

 1
 136

 1
 3

 1
 21

 2
 5

 1
 1

 1
 5

 10
 356

 1
 19

 21
 930

 2
 119

 9
 177

 2
 25

 39
 410

 2
 12

 13
 114

 13
 114

 10
 100

 2
 4

 1
 10

 17
 129

 17
 129

 1
 1

 10
 98

 4
 13

 1
 1

 1
 16

 4
 10

 1
 2

 1
 2

 3
 8

 2
 2

 1
 6

 689
 32676

 1
 1

 1
 1

 21
 303

 1
 6

 1
 7

 17
 278

 2
 12

 10
 128

 9
 126

 1
 2

 128
 4497

 126
 4473

 1
 4

 1
 20

 2
 4

 2
 4

 1
 21

 1
 21

 4
 43

 1
 19

 1
 9

 1
 9

 1
 6

 166
 3896

 2
 17

 1
 1

 1
 13

 4
 35

 1
 7

 3
 12

 8
 221

 1
 5

 72
 2335

 1
 1

 19
 662

 10
 44

 5
 48

 1
 24

 1
 2

 20
 230

 11
 120

 1
 3

 1
 7

 3
 109

 1
 1

 1
 1

 157
 20204

 3
 74

 2
 5

 1
 31

 1
 80

 13
 1039

 8
 732

 4
 705

 1
 42

 1
 4

 1
 3

 5
 969

 2
 182

 2
 31

 1
 10

 6
 2797

 5
 91

 1
 363

 1
 299

 4
 81

 6
 1154

 7
 420

 2
 275

 3
 23

 4
 718

 8
 2708

 3
 210

 4
 1844

 4
 179

 2
 347

 1
 2

 8
 84

 2
 54

 3
 1304

 3
 88

 1
 375

 22
 1660

 2
 32

 2
 1019

 8
 170

 1
 18

 1
 18

 20
 279

 3
 17

 8
 161

 3
 24

 6
 77

 125
 2062

 9
 50

 3
 25

 1
 20

 15
 129

 1
 1

 2
 10

 10
 109

 1
 15

 1
 14

 1
 6

 3
 14

 13
 119

 2
 7

 1
 5

 4
 22

 33
 1287

 1
 1

 2
 2

 9
 128

 9
 59

 4
 39

 3
 13

 3
 13

 1
 27

 1
 27

 4
 79

 4
 79

 2
 8

 2
 8

 33
 923

 18
 649

 5
 139

 10
 135

 1
 8

 1
 8

 8
 161

 2
 26

 2
 77

 3
 53

 1
 5

 139
 1422

 135
 1390

 1
 6

 6
 61

 2
 78

 5
 46

 13
 163

 1
 14

 1
 1

 1
 1

 73
 643

 1
 28

 1
 10

 27
 251

 2
 75

 1
 13

 2
 21

 2
 21

 2
 11

 1
 1

 1
 10

 44
 2156

 2
 98

 2
 98

 1
 3

 1
 3

 22
 1107

 8
 123

 8
 876

 2
 6

 1
 89

 1
 1

 1
 10

 1
 2

 1
 4

 1
 4

 3
 853

 3
 853

 6
 48

 6
 48

 1
 14

 1
 14

 5
 18

 2
 2

 1
 1

 2
 15

 3
 11

 1
 2

 1
 1

 1
 8

 127
 2661

 1
 6

 1
 6

 54
 610

 2
 11

 1
 18

 13
 79

 6
 97

 1
 2

 1
 1

 7
 207

 5
 78

 1
 4

 1
 2

 16
 111

 10
 407

 3
 346

 2
 27

 2
 7

 3
 27

 3
 6

 1
 1

 1
 1

 1
 4

 9
 156

 6
 142

 3
 14

 14
 193

 3
 44

 4
 30

 2
 23

 1
 1

 1
 68

 3
 27

 29
 1156

 23
 868

 6
 288

 5
 90

 5
 90

 2
 37

 2
 37

 98
 3145

 9
 301

 1
 65

 1
 81

 7
 155

 35
 478

 17
 148

 1
 2

 6
 176

 6
 73

 1
 71

 3
 7

 1
 1

 2
 55

 2
 55

 12
 568

 12
 568

 37
 1619

 11
 1236

 21
 369

 1
 5

 2
 2

 2
 7

 3
 124

 3
 124

 49
 630

 45
 580

 2
 7

 1
 10

 1
 1

 1
 7

 40
 555

 4
 50

 2
 37

 2
 13

 2
 35

 2
 35

 1
 1

 1
 34

 132
 2175

 1
 5

 1
 5

 131
 2170

 8
 179

 7
 158

 4
 102

 9
 88

 32
 451

 1
 5

 1
 80

 1
 1

 8
 37

 1
 7

 5
 58

 1
 3

 4
 13

 3
 10

 5
 50

 1
 1

 1
 3

 1
 24

 1
 56

 1
 25

 1
 1

 2
 133

 8
 130

 2
 24

 1
 24

 1
 6

 1
 7

 1
 4

 8
 80

 1
 9

 2
 32

 8
 369

 38
 696

 2
 21

 2
 21

 1
 1

 1
 1

 6
 66

 4
 36

 2
 30

 2
 328

 1
 294

 1
 34

 27
 280

 1
 22

 25
 257

 1
 1

 2
 3

 2
 3

 2
 3

 2
 3

 23
 143

 23
 143

 22
 136

 2
 15

 1
 10

 1
 2

 1
 11

 1
 5

 2
 8

 1
 11

 1
 4

 3
 20

 4
 11

 1
 1

 1
 7

 1
 13

 1
 11

 1
 7

 1
 7

 1
 7

 3
 8

 3
 8

 3
 8

 3
 8

 4
 12

 4
 12

 4
 12

 4
 12

 4
 12

 24
 78

 24
 78

 24
 78

 24
 78

 24
 78
